# Progress in Research on the Alleviation of Glucose Metabolism Disorders in Type 2 Diabetes Using *Cyclocarya paliurus*

**DOI:** 10.3390/nu14153169

**Published:** 2022-07-31

**Authors:** Xue Wang, Lu Tang, Wenxin Ping, Qiaofen Su, Songying Ouyang, Jingqian Su

**Affiliations:** Fujian Key Laboratory of Innate Immune Biology, Provincial University Key Laboratory of Cellular Stress Response and Metabolic Regulation, Biomedical Research Center of South China, Key Laboratory of OptoElectronic Science and Technology for Medicine of the Ministry of Education, College of Life Sciences, Fujian Normal University, Fuzhou 350117, China; wangxuexwx97@163.com (X.W.); qsx20211378@student.fjnu.edu.cn (L.T.); pwxwxwx@163.com (W.P.); 15260328242@163.com (Q.S.)

**Keywords:** *Cyclocarya paliurus*, metabolic diseases, type 2 diabetes, glucose metabolism, insulin secretion

## Abstract

Globally, the incidence of diabetes is increasing annually, and China has the largest number of patients with diabetes. Patients with type 2 diabetes need lifelong medication, with severe cases requiring surgery. Diabetes treatment may cause complications, side-effects, and postoperative sequelae that could lead to adverse health problems and significant social and economic burdens; thus, more efficient hypoglycemic drugs have become a research hotspot. Glucose metabolism disorders can promote diabetes, a systemic metabolic disease that impairs the function of other organs, including the heart, liver, and kidneys. *Cyclocarya paliurus* leaves have gathered increasing interest among researchers because of their effectiveness in ameliorating glucose metabolism disorders. At present, various compounds have been isolated from *C. paliurus*, and the main active components include polysaccharides, triterpenes, flavonoids, and phenolic acids. *C. paliurus* mainly ameliorates glucose metabolism disorders by reducing glucose uptake, regulating blood lipid levels, regulating the insulin signaling pathway, reducing β-cell apoptosis, increasing insulin synthesis and secretion, regulating abundances of intestinal microorganisms, and exhibiting α-glucosidase inhibitor activity. In this paper, the mechanism of glucose metabolism regulation by *C. paliurus* was reviewed to provide a reference to prevent and treat diabetes, hyperlipidaemia, obesity, and other metabolic diseases.

## 1. Introduction

Type 2 diabetes mellitus (T2DM) is an expanding global health problem, with the number of affected individuals increasing over the past 35 years. In 2021, approximately 537 million people were found to be affected by diabetes globally, with a prevalence of 10.5%; the number of cases is expected to reach 783 million by 2045. Further, 6.7 million out of the 537 million patients died of diabetes and its complications, accounting for ~12.2% of the total global deaths due to all causes. The global health expenditure for diabetes was estimated to be USD 966 billion in 2021. Diabetes is not only a public health threat but also imposes a significant burden on the national economy [1]. Glucose metabolism disorders are characterized by chronic hyperglycaemia and are mainly caused by defects in insulin secretion, insulin action, or both [2]. These disorders can induce the onset of diabetes, and long-term hyperglycemia can induce the development of systemic metabolic disorders that impair the function of organs, including the heart, liver, and kidneys, leading to diabetes-associated complications [3]. A high-calorie diet increases the probability of developing T2DM, and diet is one of the foundations to treat and prevent diabetes [4]. Obesity leads to increased lipid storage in the adipose tissue, and when adipose tissue can no longer store lipids, free fatty acids (FFAs) are released through increased lipolysis. Increased FFA levels promote muscle and liver insulin resistance (IR), impair insulin secretion from pancreatic β cells, attenuate glucose uptake in skeletal muscle and liver cells, increase hepatic glucose production, and decrease hepatic glycogen synthesis, ultimately leading to chronic hyperglycaemia [5]. Long-term hyperglycemia and high concentrations of fatty acids are the major causes of pancreatic β-cell failure and death [6]. Therefore, reducing appetite, regulating blood lipid levels, relieving pathologic IR, and reducing islet β-cell apoptosis could be an effective way to treat T2DM glucose metabolism disorder.

Some components of natural plants with low toxicity effectively ameliorate glucose metabolism disorders. *Cyclocarya paliurus* is a plant of the dicotyledon class Jugaceae, which is unique to China. It is widely distributed in Anhui, Jiangsu, Zhejiang, Jiangxi, Fujian, Taiwan, Hubei, Hunan, Sichuan, Guizhou, Guangxi, Guangdong, and south-eastern Yunnan and often grows in mountainous and humid forests at an altitude of 500–2500 m [7]. It is named *C. paliurus* as the tree is shaped like a willow, and its fruits are green and shaped like copper coins. Since October 2013, the National Health and Family Planning Commission of the People’s Republic of China has approved the use of *C. paliurus* leaves as a new raw food material, which has increasingly become a research hotspot because of its effectiveness in treating glucose metabolism disorders. Wang et al. reviewed the positive regulation of *C. paliurus* extract on insulin resistance, inflammation, oxidative stress, intestinal microbiota, lipid metabolism, and the protection of islet cells in type 2 diabetes with different extraction methods [8]. In our previous study, we identified 46,292 haploid genes in *C. paliurus* leaves using whole-genome sequencing. Gene Ontology and Kyoto Encyclopaedia of Genes and Genomes enrichment analysis showed that these genes are involved in polyketone biosynthesis, amino acid and nucleotide metabolism, and starch and sucrose metabolism [9]. *C. paliurus* contains various active ingredients, such as polysaccharides, triterpenes, flavonoids, and phenolic acids, present mainly in the leaves [10]. Our study revealed that the contents of polysaccharides, triterpenes, flavonoids, and phenolic acids in *C. paliurus* leaves differed in different months. Polysaccharides, triterpenes, and flavonoids were most abundant in October. In vitro studies have demonstrated that some *C. paliurus* phenolic acids, such as neochlorogenic acid and vanillic acid, exhibit α-amylase and potential hypoglycemic activities [11]. Its regulation of glucose metabolism has multiple components and targets and involves multiple channels.

This paper describes the current research status, summarizes the active components and action mechanism of *C. paliurus* in regulating glucose metabolism, and acts as a reference for resource development and the clinical application of *C. paliurus*.

## 2. Active Components of *C. paliurus*

### 2.1. C. paliurus Polysaccharide (CPP)

**Figure 1:**
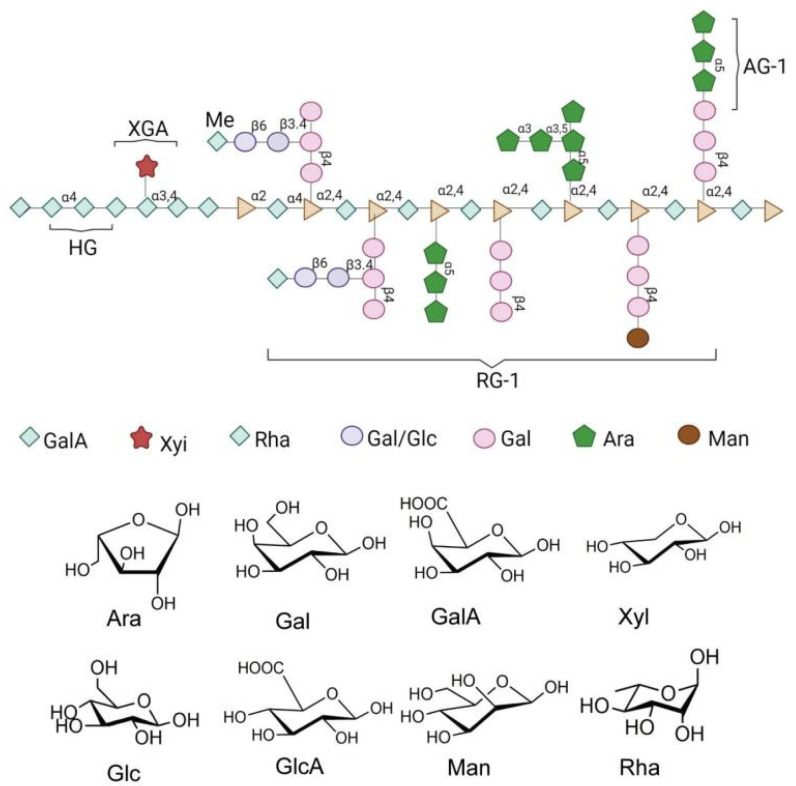
Chemical and structural formula of the *Cyclocarya paliurus* polysaccharide.

Polysaccharides are found in animals, plants, and the cell walls of microorganisms. They are safe and exhibit low toxicity when used as drugs. As an important part of our daily diet, polysaccharides are not directly digested by gastrointestinal enzymes [12]. They play a key role in maintaining human intestinal microbiota. Fermented polysaccharides produce important metabolites in the intestine, such as short-chain fatty acids (SCFAs) and succinic acid [13]. Increasing evidence has shown that polysaccharides are the main regulators of the function and composition of intestinal microbiota. Supplementing natural polysaccharides can effectively target metabolic disorders induced by a high-fat diet (HFD) by acting on the intestinal microbiota [14]. CPP, a natural macromolecule with various biological activities, is considered to be a highly effective component. As shown in Figure 1, CPP is a new pectin-like polysaccharide with high water solubility and high branching, composed of eight monosaccharides: galacturonic acid, glucose, galactose, arabinose, mannose, xylose, rhamnose, and glucuronic acid [15]. As shown in Table 1, in vivo experiments have shown that CPP can alleviate the symptoms of T2DM and ameliorate glucose and lipid metabolism disorders in T2DM rats by regulating intestinal microbiota and SCFA levels [8,16,17,18,19].

### 2.2. C. paliurus Flavonoids

Flavonoids are considered to be a class of biologically active plant secondary metabolites that have antiviral, anti-allergic, antibacterial, and anti-inflammatory functions [27]. Flavonoids are composed of 15 carbon skeletons and two aromatic rings (A and B), which are connected by a three-carbon chain [28]. Several studies have shown that flavonoids exert various positive effects on metabolic disorders. The regulatory functions of flavonoids in promoting carbohydrate digestion, insulin signal transduction, insulin secretion, glucose uptake, and fat deposition facilitate the potential anti-T2DM activity [29]. As described in Table 2, previous studies have shown that *C. paliurus* flavonoids ameliorate liver lipid deposition in rats [30], regulate intestinal microorganisms in obese mice [31], reduce blood glucose levels [32], and act as antioxidants [33].

### 2.3. C. paliurus Triterpenes

Triterpenes are mainly found on plant surfaces [42]. Pentacyclic triterpenoids and their derivatives inhibit α-glucosidase, α-amylase, and pancreatic lipase activity, regulating blood glucose levels and reducing insulin resistance. They have become one of the most widely studied anti-diabetic compounds [43]. The total triterpenoid content of *C. paliurus* has also been shown to ameliorate glucose and lipid metabolism disorders [20]. A total of 137 triterpenoids are found in *C. paliurus* leaves [10], as described in Table 2, many of which promote glucose uptake [21,44,45] and improve lipid metabolism [46,47].

### 2.4. C. paliurus Phenolic Acids

Phenolic acids are widely found in fruits, vegetables, grains, beans, wine, and dairy foods, which have been shown to increase glucose intake and glycogen synthesis and ameliorate glucose and lipid metabolism disorders in obesity, cardiovascular disease, and diabetes and its complications [48]. As described in Table 2, *C. paliurus* phenolic acids also have a certain antioxidant activity shown by both in vivo and in vitro studies [49].

## 3. Mechanism of Regulating Glucose Metabolism

### 3.1. Reduction of Glucose Intake

Excessive nutritional intake is closely associated with insulin resistance [50], which can cause a series of pathological reactions, including hyperinsulinemia, β-cell apoptosis, and oxidative stress, finally leading to T2DM. The hypothalamus is the main center in the brain that regulates appetite and plays an essential role in regulating energy intake [51]. The hypothalamic insulin signaling pathway is also involved in the regulation of appetite [52]. The hypothalamic expression levels of proopiomelanocortin (POMC) and neuropeptide Y (NPY) are closely associated with the control of food intake. The upregulation of POMC reduces appetite, as does the downregulation of NPY [53]. The phosphoinositide 3-kinase (PI3K)-dependent activation of protein kinase B (Akt) can inhibit the activity of Forkhead box 1 (FOXO1) [54], which stimulates NPY transcription and inhibits POMC transcription [55].

**Figure 2:**
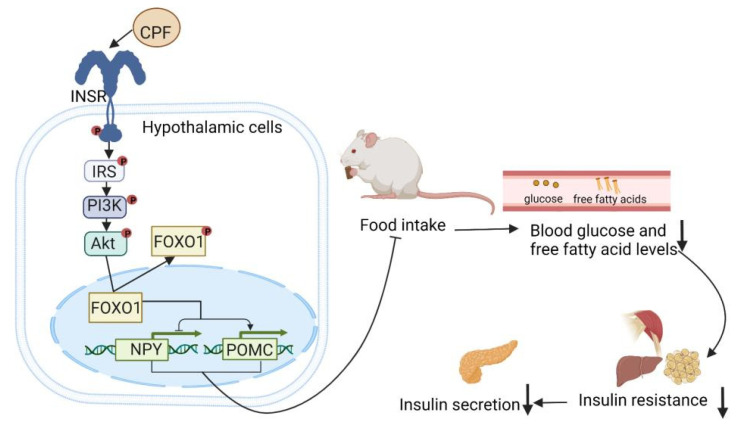
The mechanism of action of *Cyclocarya paliurus* flavonoids with respect to decreasing appetite. ↑: increase; ↓: decline.

As shown in Figure 2, *C. paliurus* flavonoids have been demonstrated to reduce food intake, body weight, serum insulin level, and insulin resistance in SHR/CP rats. this may be due to the increased phosphorylation of the insulin receptor (INSR)/insulin receptor substrate-1 (IRS1)/PI3K/Akt/FOXO1 pathway, thereby stimulating the hypothalamic insulin signal, which, in turn, regulates appetite and reduces food intake by regulating the expression of NPY and POMC [24].

### 3.2. Blood Lipid Level Reduction

Obesity induces increased lipid storage in adipose tissues. When the adipose tissues no longer have the capacity to store lipids, FFAs are released through increased lipolysis. Once the FFAs enter the blood, they are deposited on the lining of blood vessels, leading to increased blood lipid levels. Increased FFA levels promote muscle and liver IR [5]. Long-term hyperglycaemia and high FFA levels are major causes of pancreatic β-cell failure and death [6]; thus, regulating blood lipid levels is of great significance in glucose metabolism.

#### 3.2.1. Inhibition of Fatty Acid Synthesis or Lipogenesis

**Figure 3:**
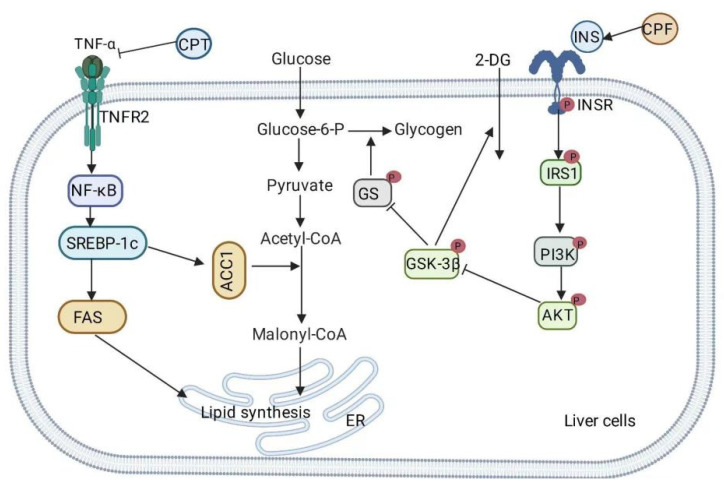
The mechanism of action of *Cyclocarya paliurus* extract with respect to inhibiting lipid synthesis and alleviating pathological insulin resistance in liver cells.

Tumor necrosis factor-α (TNF-α) stimulates hepatic lipid deposition by enhancing the expression of the sterol regulatory element-binding protein 1c (SREBP-1c) gene [56]. SREBP-1c plays an important role in hepatic lipogenesis by regulating the expression of its downstream genes, such as fatty acid synthase (FAS) and acetyl-CoA carboxylase 1 (ACC-1) [57]. Lin et al. induced hepatic steatosis in Sprague–Dawley rats by feeding them an HFD. They found that *C. paliurus* triterpenoids decreased mRNA expression levels; blood lipid levels; TNF-α, SREBP1, ACC1, and FAS protein levels in the HFD-fed Sprague–Dawley rats. Moreover, hepatic fat deposition improved, and hepatic fat content reduced (Figure 3) [23].

#### 3.2.2. Inhibition of Exogenous Lipid Uptake

**Figure 4:**
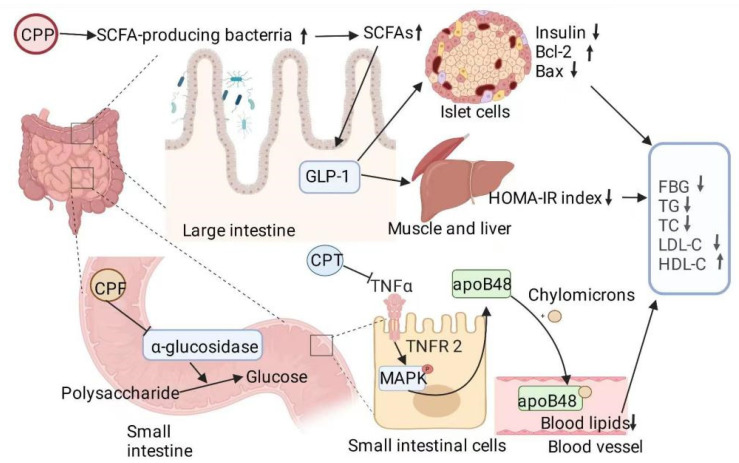
The mechanism of action of *Cyclocarya paliurus* extract with respect to lowering blood glucose and lipid levels through intestinal metabolism. ↑: increase; ↓: decline.

Apolipoprotein B48 (apoB48) is a component protein of chylomicrons and participates in dietary lipid absorption [58]. ApoB48 promotes the absorption of exogenous lipids in the intestine, and, thus, apoB48 overexpression may lead to hyperlipidaemia [59]. TNF-α, a major pro-inflammatory cytokine, also plays a key role in lipid metabolism, especially lipid absorption [60]. TNF-α stimulates the activation of the p38 mitogen-activated protein kinase (MAPK) pathway and promotes the production of lipoproteins that contain the apoB48 protein in the intestine [61]. Ethanolic extracts and triterpenes from *C. paliurus* inhibit TNF-α-induced MAPK phosphorylation and apoB48 production and reduce the levels of triacylglycerol (TG), total cholesterol (TC), high-density lipoprotein-C (HDL-C), and low-density lipoprotein-C (LDL-C) in the blood, thereby conferring an anti-hyperlipidemic effect (Figure 4) [22,62].

### 3.3. Regulation of the Insulin Signaling Pathway

**Figure 5:**
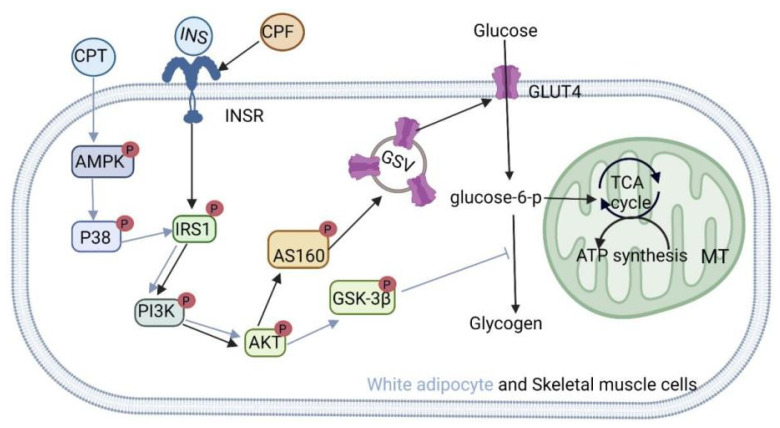
The mechanism of action of *Cyclocarya paliurus* extract with respect to inhibiting pathological insulin resistance in white adipocytes and muscle cells.

The insulin signaling pathway can be roughly summarized as follows: insulin binds to the INSR and activates the IRS by increasing its tyrosine residue phosphorylation. The IRS then regulates PI3K/Akt phosphorylation, which, in turn, regulates blood glucose. A defect in the pathway can produce pathologic IR [63]. IR is a systemic metabolic disorder characterized by hypoinsulinemia in the skeletal muscle, liver, and adipocytes [64]. IR and the consequent hyperinsulinemia lead to increased pancreatic β-cell apoptosis and decreased β-cell mass [65]. Obesity-related IR is closely associated with overnutrition, which overdrives the processes of nutrient utilization (endoplasmic reticulum (ER) stress and oxidative stress) or cytotoxic response (inflammation) mediated by nutritional stress [48]. Based on current research, various active components in *C. paliurus* improve insulin signal transduction in multiple tissues and cells, such as muscle tissue, adipocytes, and liver cells. As shown in Figure 5, Fang et al. found that *C. paliurus* triterpenoids enhance the phosphorylation of key proteins (IRS-1, Akt, and glycogen synthase kinase β (GSK-3β)) in the insulin signaling pathway and that insulin stimulates glucose uptake in fully differentiated 3T3-L1 adipocytes [21]. *C. paliurus* flavonoids activate insulin signaling in an insulin-independent manner (IRS1-PI3K-Akt-AS160 (Tbc1d family Rab GTPase activator)), which brings about increased glucose uptake by inducing the translocation of glucose transporter 4 (GLUT4) in C2C12 cells. The plasma glucose levels of streptozocin (STZ)-induced hyperglycemic mice were moderately reduced 1 h after a single administration of *C. paliurus* flavonoids, with increased AS160 and Akt phosphorylation in skeletal muscles (Figure 5) [66].

Zheng et al. found that *C. paliurus* triterpenoids remarkably reduced the levels of serum and liver TG and TC in C57BL/6J mice that were fed an HFD. Furthermore, they significantly reduced blood glucose and insulin levels, reduced the IR index (HOMA-IR), increased the tyrosine residue phosphorylation level of IRS and 2-deoxyglucose (2DG) uptake in palmitic acid-induced HepG2 cells and primary hepatocyte fatty liver models, and reduced the number of lipid droplets and intracellular TG content. A mechanistic study showed that *C. paliurus* triterpenoids increased the phosphorylation of PI3K, Akt, and GSK-3β (Figure 3) [20].

### 3.4. Reduction of β-Cell Islet Apoptosis

Studies have shown that both high glucose levels and high concentrations of FFAs induce a stress response in pancreatic β cells, including ER stress and mitochondrial dysfunction caused by oxidative stress secondary to excessive reactive oxygen species (ROS) production and inflammation. Crosstalk between these pathways may induce a feed-forward mechanism and aggravate toxic glycolipid stress, which could eventually lead to β-cell dysfunction, apoptosis, and possible dedifferentiation [67].

β cells are predisposed to ER stress because of the high demands on the ER incurred by insulin synthesis, especially during IR, which requires large amounts of insulin to be produced. This leads to an increased expression of ER stress markers and β-cell ER expansion in patients with T2DM. The ER plays a critical role in lipid biosynthesis, Ca^2+^ storage, and synthesis and folding of secreted proteins. The accumulation of unfolded or misfolded proteins in the ER results in ER stress, which activates the unfolded protein response [68].

This set of intracellular signaling pathways is initiated by three ER stress sensors and transducers, specifically, protein kinase RNA-like endoplasmic reticulum kinase (PERK), activating transcription factor-6 (ATF6), and inositol requiring enzyme-1 (IRE1). The purpose of signal transduction is to restore ER homeostasis by deadening overall protein translation, upregulating ER folding capacity, and reducing misfolded proteins [68,69]. The apoptosis will be triggered if the goal is not achieved [70].

β cells are also highly sensitive to oxidative stress. ROS, which act as important signaling messengers, are generated in these cells during glucose metabolism to trigger insulin secretion and β-cell expansion in response to elevated glucose levels. However, prolonged exposure to ROS under hyperglycemic conditions results in cellular damage, impaired glucose-stimulated insulin secretion, and ultimately, β-cell death [71]. The ROS produced by β cells in response to metabolic stress affect mitochondrial structure and function. Specifically, ROS oxidize mitochondrial membrane phospholipids such as cardiolipin and impair membrane integrity, which results in the release of cytochrome c, thereby triggering apoptosis [70]. In a study that combined STZ with HFD-induced diabetic mice and high-sugar-fed and HFD-fed mice, treatment with *C. paliurus* triterpenoids significantly reduced the serum malondialdehyde content (MDA), reduced oxidative stress, and enhanced the activities of the antioxidant enzymes superoxide dismutase (SOD) and glutathione peroxidase (GSH-Px). In the STZ-induced NIT-1 cell injury model, *C. paliurus* triterpenoids significantly reduced ROS production and cell apoptosis, promoted cell proliferation, and alleviated STZ-induced damage to NIT-1 cells [72].

**Figure 6:**
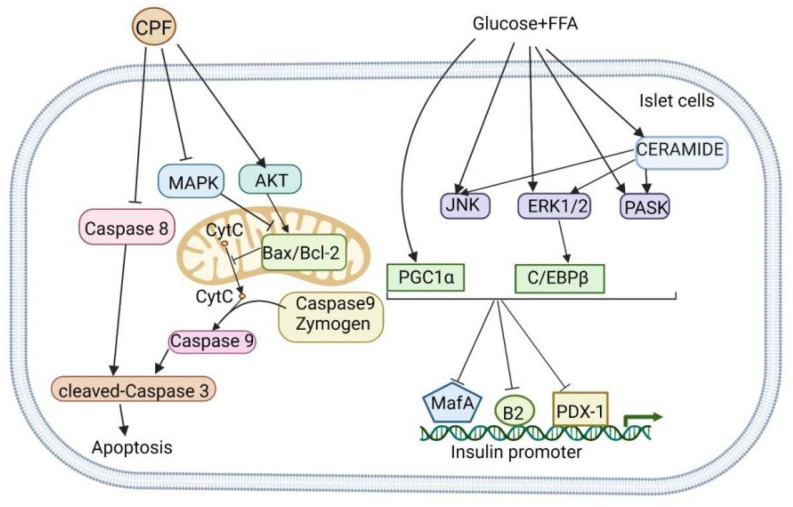
The mechanism of action of *Cyclocarya paliurus* flavone with respect to alleviating islet cell apoptosis and relatively insufficient insulin secretion caused by glycolipid toxicity.

Akt kinases and kinases in the MAPK superfamily play important roles in regulating pancreatic β-cell apoptosis [6]. Activation of the PI3K/AKT pathway upregulates the expression of the Bax/Bcl-2 complex [73], while activation of the MAPK pathway downregulates the expression of the same complex [74], and apoptosis can be invited by intrinsic or extrinsic pathways. The intrinsic pathway activates internal signals through Bax, Bcl-2, cytochrome c, and caspase-9. The Bax protein also has an inhibitory effect on Bcl-2, an antiapoptotic protein. Endogenous apoptosis occurs when the Bax protein is stimulated to move to and from an opening in the outer membrane. Cytochrome c migrates from the mitochondrial membrane into the cytoplasm to trigger the formation of apoptotic bodies, which activates caspase-9 and ultimately promotes cell death; the activation of caspase-8 in the exogenous pathway leads to apoptosis [75]. Xiao et al. found that the aqueous extract of *C. paliurus* increased the number of pancreatic β cells by decreasing β-cell apoptosis in mice in which diabetes was induced by feeding an HFD combined with STZ treatment. Using STZ to induce NIT-1 cells to establish an in vitro apoptosis model of pancreatic β cells, they found that the aqueous extract of *C. paliurus* inhibits NIT-1-cell apoptosis. Studies have shown that the aqueous extract of *C. paliurus* downregulates the phosphorylation of p38, ERK, and c-Jun N-terminal kinase (JNK). It can also upregulate Akt phosphorylation; inhibit the expression of caspase-8, caspase-9, and cleaved caspase-3; decrease the Bax/Bcl-2 ratio, all of which significantly inhibit pancreatic β-cell apoptosis (Figure 6) [25].

The abovementioned stress pathways interact with each other. Glucose metabolism produces excess ROS in a high-glucose environment, which induces Ca^2+^ consumption in the ER, bringing about ER stress [76]. ER stress induces the expression of pro-inflammatory genes by activating transcription factors such as NF-κB and JNK [77]. Mild ER stress sensitizes β cells to the IL-1β and TNF-α cytokines, which amplify the inflammatory response [78]. At present, only a few studies have reported the inhibition of β-cell apoptosis by *C. paliurus* through the above stress pathways.

### 3.5. Promotion of Synthesis and Secretion of Insulin

Insulin release from pancreatic β cells is a highly coordinated process involving insulin gene transcription, proinsulin biosynthesis, and insulin secretion. As the major insulin secretagogue, glucose actively regulates the insulin release to ensure adequate intracellular stores and fulfill the secretion demands [79,80,81]. Lipotoxicity and glucolipotoxicity have been exhibited to alter several key functions of insulin secretion. High glucose and FFA palmitate levels stimulate ceramide production [3,4] and activate the stress kinases JNK [82], extracellular regulated protein kinases (ERK1/2), and PAS domain-containing serine/threonine kinase (PASK) [83], partly through the activity of the transcription factor peroxisome proliferator-activated receptor-γ coactivator-1α (PGC-1α) and CCAAT enhancer-binding proteins (C-EBPβ) (Figure 6). These proteins inhibit the binding of the transcriptional regulators musculoaponeurotic fibrosarcoma oncogene homolog A (MafA), insulin promoting factor-1 (PDX-1), and neurogenic differentiation (NeuroD) to the insulin promoter [84,85], which ultimately brings about decreased insulin transcription levels. Glucose triggers insulin secretion by producing numerous coupling factors, including changes in the adenosine triphosphate (ATP)/adenosine triphosphate (ADP) ratio, closure of the ATP-sensitive potassium channels, and depolarization of cell membranes. Calcium influx stimulates insulin exocytosis, and insulin secretion is affected, to varying degrees, by exposure to chronic FFA environments [70].

A few studies have reported the induction of insulin synthesis and secretion by *C. paliurus* extract. Feng et al. found that the flavonoids in *C. paliurus* enhances insulin secretion stimulated by glucose in normal MIN6 cells [86]; nevertheless, the specific mechanism has not yet been elucidated.

### 3.6. Regulation of Intestinal Microorganisms

The 16S rRNA high-throughput sequencing technology and bioinformatic analysis has developed by leaps and bounds, and scientists have realized that the intestinal microbiota is essential to human health. Recent studies have found that imbalances in the composition of intestinal microbiota are critical for metabolic diseases [87] and that the intestinal microbiota plays a critical role in metabolic diseases, notably those combined with low-grade chronic inflammation [88].

The intestinal microbiota both affects the digestion and absorption of food and energy supply and regulates the physiology of the host and development of diseases. The physiological metabolism of the body is regulated by genes and intestinal microbiota [89]. Daily diet can influence host susceptibility to most chronic diseases by altering intestinal microbiota composition, metabolites, and metabolic functions [90]. As shown in Figure 4, Wu et al. found that CPPs increased the number of SCFAs (acetic acid, propionic acid, butyric acid, and valeric acid) in the feces of healthy mice in a dose-dependent manner. Sequencing of 16S rRNA showed that the administration of 200 mg/kg of CPPs effectively increased intestinal microbiota diversity in healthy mice as well as affected the relative abundance of *Spirillum* and *Clostridium* sp., enhancing the metabolic function of the intestinal microbiota [18]. Obesity and metabolic syndrome are associated with a decrease in the abundance of *Clostridium*. The presence of *Clostridium* brings about reduced lipid absorption and body emaciation by downregulating expression of CD36, a receptor that mediates the binding and uptake of long-chain fatty acids [91]. Meehan et al. found that Lachnospiraceae, a member of the order Clostridiales, can protect against obesity and colon cancer in humans by producing butyrate [92]. Yao et al. found that CPPs can alleviate the symptoms of T2DM by increasing the abundance of SCFA-producing bacteria, promoting SCFA production, and increasing the production of SCFA-GLP1/PYY-related sensory mediators in STZ- and HFD-induced diabetic rats [17]. Li et al. found that the intragastric administration of CPPs increased the abundance of *Ruminococcus* UCG-005, a beneficial bacterium, in STZ- and HFD-fed diabetic rats, increase the level of serum GLP1 and the content of intestinal SCFAs, and reduce the blood glucose, serum insulin and Bax/Bcl-2 ratio of rats. UCG-005 is the key bacterium that prevents the onset of diabetes by promoting fecal SCFA production. The analysis of urine metabolites showed that CPP treatment helped protect against diabetes by significantly enhancing the activity of several pathways closely associated with nutritional (amino acid and purine) and energy metabolism (tricarboxylic acid cycle) [16].

### 3.7. Inhibition of α-Glucosidase Activity

α-Glucosidase is a glycoside hydrolase that is responsible for hydrolyzing disaccharides. It is essential for absorbing starch, dextrin, and disaccharides. Inhibition of α-glucosidase causes malabsorption and slower carbohydrate absorption, which attenuates the increase in postprandial blood glucose levels. α-Glucosidase inhibitors may also increase the release of glucagon-like peptide-1 (GLP-1), contributing to the hypoglycemic effect. α-Glucosidase inhibitors effectively improve glycemic control in T2DM [93]. Li et al. extracted more than 20 triterpenoids from the aqueous extract of *C. paliurus*, of which cyclocarioside Z9 and cyclocarioside Z10 strongly inhibited the α-glucosidase activity with IC_50_ values of 257.74 μM and 282.23 μM, respectively. The IC_50_ value of acarbose, a positive control drug, was 359.36 μM [46]. Ning et al. found that the aqueous extract of *C. paliurus* had a significant α-glucosidase inhibitory activity, with an IC_50_ value of 31.5 ± 1.05 μg/mL, which was much lower than that of the positive control acarbose (IC_50_ = 296.6 ± 1.06 μg/mL). Ultra-performance liquid chromatography and quadrupole time-of-flight mass spectrometry and an in vitro α-glucosidase inhibition test were used to identify and confirm that kaempferol-3-o-rhamnoside, quercetin, kaempferol, asiatic acid, and genistein were the main components that inhibited α-glucosidase activity in the *C. paliurus* extract. In vitro experiments also confirmed that these α-glucosidase inhibitors reduce postprandial hyperglycaemia in C57BL/6J mice (Figure 4) [26].

## 4. Conclusions

*C. paliurus* is a healthy food resource that is unique to China. It has a significant hypoglycemic effect, high economic value, and great development and application potential. The causes of diabetes are complex, and a single preventive and treatment approach has limited effects and does not provide an effective therapeutic outcome. The main functional components of *C. paliurus* involved in glucose metabolism regulation are polysaccharides, triterpenes, and flavonoids, which can be used to treat glucose metabolism disorder in diabetes by reducing glucose uptake, regulating blood lipids, regulating insulin signaling pathways, and reducing β-cell apoptosis. Furthermore, these components increase insulin synthesis and secretion, regulate the abundance of intestinal microorganisms, and inhibit α-glucosidase activity.

*C. paliurus* is widely distributed, and the main components vary greatly with the areas of production, solvent polarity, or extraction methods. As a new raw food material, the composition and content of its active components need to be further elucidated. However, *C. paliurus* components are complex and diverse, and the regulation of glucose metabolism involves multiple targets and channels. Moreover, most of the mechanistic studies in this review were conducted using extracts, while few studies were conducted on monomeric components. Therefore, animal experiments or clinical verification is in urgent need to explore the specific molecular mechanisms of the *C. paliurus* components. In addition, although many animal experiments have shown that the *C. paliurus* extract ameliorates effects of glucose metabolism disorders, there is a lack of clinical trial data to support the findings.

Further studies are recommended on the following aspects. First, the number of population samples for food test research should be increased to the maximum to guide the development and utilization of *C. paliurus* as a functional food. Second, using advanced technologies such as transcriptomics and metabolomics, the specific mechanism of *C. paliurus* in regulating glucose and lipid metabolism should be further explored. Lastly, the material basis of the function of *C*. *paliurus* should be studied in detail to develop products with a controllable and standardized composition and content, to determine the optimal dose for biological activity.

In this review, the mechanism of action of the *C. paliurus* extract in treating glucose metabolism disorders in diabetes was reviewed. With more in-depth studies on the chemical constituents of *C. paliurus*, new pharmacological effects will be discovered. With the aid of developments in science and technology, we will further study the complete mechanistic network of *C. paliurus* in diabetes treatment with attention to the relationship between its efficacy and side effects.

## Figures and Tables

**Table 1:** The mechanism of action of the different *Cyclocarya paliurus* extracts on type 2 diabetes mellitus.

Active Ingredient	Extraction Method	Extraction Conditions	Biological Function	Possible Mechanism	Model		Reference
					In Vivo	In Vitro	
Polysaccharide	Water extraction	Dry leaves powder; Solid residue (hot water); Crude polysaccharide (dialysis, protein removal, decolorization chromatographic column); Purified polysaccharide.	Regulation of intestinal microorganisms	SCFAs→GLP-1→Insulin signaling pathway	Wistar rats High-fat feed + 30 mg/kg STZ		[16]
Polysaccharide	Alcohol extraction	Dry leaves with 80% ethanol for 24 h; Residue (dry, soak in distilled water, filter); Filtrate (decompression, concentration); Filtrate (add anhydrous ethanol, centrifuged); C. paliurus polysaccharide.	Regulation of intestinal microorganisms	SCFAs→SCFAs-GLP1/PYY	SD rats High-fat feed + 35 mg/kg STZ		[17]
Triterpene	Alcohol extraction	Dry leaves with 80% ethanol for 24 h; Extractive (decompression, concentration, degreased with petroleum ether, partitioned with chloroform); Triterpenic acid-rich fraction.	Improvement of insulin resistance	PI3K→Akt→ GSK3β	C57BL/6J mice High-fat feed	HepG2 cells induced by 100 mM PA	[20]
Triterpene	Alcohol extraction	Dry leaves with 70% EtOH at room temperature; Crude extract suspended in H_2_O, successively partitioned with petroleum ether, EtoAc, and n-BuOH.	Improvement of islet resistance	AMPK→P38→ PI3K→Akt→ GSK3β		C2C12 cells 1% P/S, differentiation induced by 2% heat-inactivated HS 3T3-L1 cells Differentiation was induced by 1 µM dexamethasone, 0.5 mM 3-isobutyl-1-methylxanthine, and 5 µg/mL insulin	[21]
Triterpene	Alcohol extraction	Dry leaves with 80% alcohol; Extractive (decompression, concentration, degreased with petroleum ether, partitioned with chloroform); Chloroform fraction (dissolved in chloroform, partitioned with NaOH, neutralizing the aqueous phase with HCl, re-extracted with chloroform); Triterpenic acid-rich fraction.	Hypolipidemia	TNF-α→MAPK→apoB48	SD rats High-fat feed		[22]
Triterpene	Alcohol extraction + chloroform extraction	Dry leaves with 80% ethanol; Extractive (decompression, concentration, partitioned with chloroform); *C. paliurus* chloroform extract.	Hypolipidemia	SREBP→ACC1/ FAS→Lipid synthesis	SD rats High-fat feed		[23]
Flavone	Water extraction	Dry leaves with 80% ethanol; Extractive (decompression, concentration, degreased with petroleum ether); Freeze-dried after decompression and concentration. Crude extract.	Reduction in appetite	PI3K/Akt→ FOXO1t→POMC/NPY	SHR/cp rats		[24]
Flavone	Water extraction	Dry leaves boiled with water; Aqueous extract (concentrated and dried under reduced pressure); Crude extract.	Inhibition of islet cell apoptosis	p38→ERK→JNK→Akt	C57BL/6J mice High-fat feed + 25 mg/kg (3 day)	NIT-1 cells 11.1 mM glucose	[25]
Flavone	Water extraction	Dry leaves boiled with water; Aqueous extract (concentrated and dried under reduced pressure); Crude extract.	Inhibition of the α-glucosidase activity		C57BL/6J mice		[26]

Abbreviations: NPY, neuropeptide Y; POMC, proopiomelanocortin; SCFAs, short-chain fatty acids.

**Table 2:** Therapeutic effects of small molecules in *Cyclocarya paliurus* on type 2 diabetes mellitus.

Classification	Name (9)	Structure (9)	Relevant Indicators	Model		Reference
				In Vivo	In Vitro	
Flavone	Fisetin	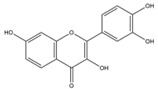	FBG, Serum insulin, Glycosylation of red cells	Wistar rats 50 mg/kg STZ		[34]
	Kaempferol	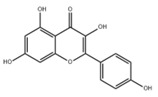	FBG, GSK3β phosphorylation, Liver pyruvate carboxylase activity, gluconeogenesis	C57BL/6 mice 40 mg/kg STZ (3 days)		[35]
	Quercetin	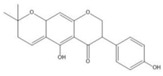	FBG, Pancreatic iron deposition, Pancreatic PBC iron death, ROS, Serum insulin	C57BL/6 mice High-fat feed + 50 mg/kg STZ		[36]
	Isorhamnetin	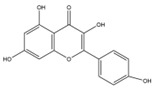	FBG, Serum insulin, HOMA-IR, BW, blood fat, LncRNA-RP11-773H22.4	Wistar rats High-fat feed + 30 mg/kg STZ		[37]
	Naringenin	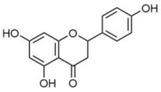	FBG, BW, Impaired glucose tolerance, Serum insulin, HOMA-IR, ROS	C57BLKsJ db^/+^ mice		[38]
	Apigenin	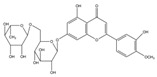	Renal injury, Pro-inflammatory gene expression, CD38, Sirt3, ROS	Zucker rats		[39]
Triterpene	Oleanolic acid	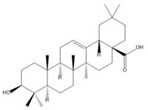	BW, Liver weight, LDL, TG, SREBP, total bilirubin, Liver injury	SD rats High-sugar and high-fat feed		[40]
	Maslinic acid	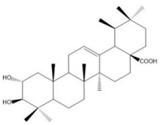	BW, FBG, food intake, urinary albumin, Renal injury, kidney ROS	C57BL/6 mice 50 mg/kg STZ		[41]
	(20S,24R)-20,24-Epoxy-25-hydroxy-12β-(α- l -arabinopyranosyloxy)-3,4-seco-dammara-4(28)-en-3-oic acid	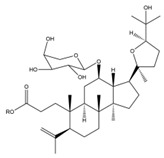	AMP (AMPK)-p38 access, Glucose uptake		C2C12 cells differentiation induced by 1% P/s and 2% heat-inactivated horse serum 3T3-L1 cells differentiation was induced by 1 µM dexamethasone, 0.5 mM 3-isobutyl-1-methylxanthine, and 5 µg/mL of insulin	[21]

Abbreviations: BW, body weight; FBG, fasting blood glucose; HOMA-IR, insulin resistance index; LDL, low-density lipoprotein; PBC, pancreatic β cells; ROS, reactive oxygen species; SD, Sprague–Dawley; STZ, streptozocin; TG, triacylglycerol.

## Abbreviations

2-DG: 2-deoxyglucose; ACC-1, acetyl-CoA carboxylase 1; ATF6, activating transcription factor-6; ALT, alanine aminotransferase; AST, aspartate aminotransferase; BW, body weight; CAT, catalase; ERK, extracellular regulated protein kinases; FAS, fatty acid synthase; FBG, fasting blood glucose; FFA, free fatty acids; GSH-Px, glutathione peroxidase; HDL-C, high-density lipoprotein-C; HFD, high-fat diet; HOMA-IR, IRE1, inositol requiring enzyme-1; insulin resistance index; INSR, insulin receptor; IR, insulin resistance; IRS1, insulin receptor substrate-1; LDL, low-density lipoprotein; LDL-C, low-density lipoprotein-C; MDA, malondialdehyde; NeuroD, neurogenic differentiation; NPY, neuropeptide Y; OGTT, oral glucose tolerance test; PASK, PAS domain-containing serine/threonine kinase; PBC, pancreatic β cells; POMC, proopiomelanocortin; PERK, protein kinase RNA-like endoplasmic reticulum kinase; ROS, reactive oxygen species; SCFAs, short-chain fatty acids; SD, Sprague–Dawley; SREBP-1c, sterol regulatory element-binding protein 1c gene; SOD, superoxide dismutase; STZ, streptozocin; TC, total cholesterol; TG, triacylglycerol.
